# Epidemiological analysis of syphilis trends, disparities, and public health implications in the United States, 2018–2022

**DOI:** 10.1186/s12879-025-11332-4

**Published:** 2025-09-12

**Authors:** Erica Rankin, Ashley Forrest, Lahana Maharjan, Griffin Wei, Cyril Blavo, Jyotsna Chawla

**Affiliations:** 1 Nova Southeastern University, Dr. Kiran C. Patel College of Osteopathic Medicine, Fort Lauderdale, FL USA; 2 Nova Southeastern University, Dr. Kiran C. Patel College of Osteopathic Medicine, Tampa, FL USA; 3 International Health Initiatives, Inc, Davie, FL USA; 4 Washington University in St. Louis, St. Louis, MO USA

**Keywords:** Syphilis, Sexually transmitted infections (STIs), Epidemiology, Health disparities, Behavioral risk factors, CDC surveillance data

## Abstract

Syphilis has experienced a marked resurgence in the United States since 2018, with incidence rates reaching levels not observed since the pre-antibiotic era. Despite growing awareness among healthcare professionals and public health authorities, a comprehensive epidemiological analysis of the populations most affected during this period remains limited. This study examines the rise in syphilis cases from 2018 to 2022 using surveillance data from the Centers for Disease Control and Prevention (CDC). We evaluate trends across key demographic variables including race, sexual behavior, geographic distribution, substance use, and sex of sexual partner. Additionally, we assess the role of socioeconomic determinants that may impede access to prevention, diagnosis, and treatment. By identifying disproportionately affected populations, this research aims to inform targeted public health interventions and guide evidence-based policy responses to mitigate the ongoing syphilis epidemic.

**Supplementary Information:**

The online version contains supplementary material available at 10.1186/s12879-025-11332-4.

## Introduction

Syphilis, one of the most historically significant and devastating sexually transmitted infections (STIs), reached its peak prevalence in the United States in 1947. This burden sharply declined following the widespread availability of penicillin, a breakthrough that transformed the landscape of infectious disease control [1]. For decades thereafter, syphilis rates steadily declined and eventually plateaued at relatively low levels by the early 2000 s, stabilizing around 11 to 12 cases per 100,000 population [2]. However, in recent years, a troubling reversal has emerged. Since 2018, the United States has experienced a dramatic resurgence in syphilis cases, including a striking 80% increase in newly reported infections [3]. Despite the availability of effective diagnostic tools, the mechanisms driving this sharp increase remain insufficiently understood and underexplored in the current literature.

Syphilis, caused by the motile spirochete *Treponema pallidum*, is transmitted primarily through sexual contact (vaginal, anal, or oral) or vertically during pregnancy, leading to congenital syphilis with severe outcomes like stillbirth [4]. The bacterium invades via mucous membranes or microscopic abrasions, with an incubation period of 3 to 4 weeks before a primary chancre appears. Often termed “the great imitator” due to its highly variable symptoms mimicking conditions such as viral illnesses, skin disorders like psoriasis or pityriasis rosea, neurological diseases including stroke or dementia, and autoimmune conditions, syphilis poses significant diagnostic challenges, often leading to delayed or missed diagnoses. Primary syphilis presents with a painless chancre and regional lymphadenopathy, while secondary syphilis may involve fever, malaise, diffuse rash (often affecting palms and soles), mucous patches, or condylomata lata. If untreated, latent syphilis can persist for years, with up to one-third of cases progressing to tertiary syphilis, causing severe cardiovascular, neurological, or gummatous complications, such as destructive skin, bone, or visceral lesions [1, 5]. Recent surges in syphilis incidence, including ocular and neurosyphilis, underscore its evolving epidemiology and the need for heightened clinical vigilance.

Given the severe consequences of untreated syphilis and its reemergence as a significant public health concern, this study investigates the epidemiological trends of syphilis in the United States between 2018 and 2022 using national surveillance data. Specifically, we aim to characterize patterns of syphilis prevalence across racial and ethnic groups, sexual behaviors, geographic regions, and substance use patterns. A deeper understanding of the epidemiological and behavioral factors associated with rising syphilis rates is essential for developing targeted public health interventions and addressing persistent disparities. Insights from this analysis may help guide more effective resource allocation and inform strategies to reduce the ongoing spread of syphilis in the United States.

## Methods

### Data source and surveillance system

This study utilized publicly available data from the Centers for Disease Control and Prevention (CDC) national surveillance system STIs), which provides comprehensive epidemiological data on infectious disease trends in the United States from 1941 to 2022. The CDC dataset is compiled through the National Notifiable Disease Surveillance System (NNDSS), a standardized reporting system for tracking and monitoring infectious diseases nationwide. This database includes case counts and rates for chlamydia, gonorrhea, syphilis, and congenital syphilis, reported annually by state and local health departments, as well as age, sex and gender, sexual behaviors practiced, sex of reported partner, substance use information, and geographic location.

### Data extraction and variable selection

1. For the purpose of this study, we extracted data specifically related to syphilis, including primary and secondary syphilis, as well as congenital syphilis. Syphilis data were further stratified and analyzed across seven key variables: STI Trends: Percent changes in cases of chlamydia, gonorrhea, and syphilis over one-year (2021–2022) and five-year (2018–2022) periods.
2. Race/Ethnicity: Syphilis rates per 100,000 population were evaluated for the following racial and ethnic groups: American Indian/Alaska Native (AI/AN), Asian, Black/African American (AA), Hispanic/Latino, Multiracial, Native Hawaiian/Pacific Islander (NH/PI), and White.
3. Sexual Behavior: Analysis of reported behaviors, including exchanging sex for drugs or money, engaging in sex while intoxicated and/or high on drugs, sex with anonymous partners, and sex with a person who injects drugs.
4. Sex of Partner: Cases of syphilis were stratified based on reported sex of sexual partner, categorized as: men who have sex with men (MSM), men who have sex with women only (MSW), men with unknown sex of partners (MSU), and women.
5. Substance Use: Patterns of substance use were analyzed, including cocaine, crack, heroin, injection drug use, and methamphetamine use.
6. Sex and Gender: Syphilis cases were assessed based on biological sex (male and female) from 2018 to 2022.
7. Age Groups: Rates of syphilis were evaluated for males and females across age groups (15–19, 20–24, 25–29, 30–34, and 35–44 years), with rates averaged over the five-year study period.
8. Geographic Distribution: States with the highest reported rates of primary and secondary syphilis in 2022 were identified and visualized.

### Statistical analysis

Descriptive analysis was conducted to examine trends in primary and secondary syphilis across demographic, behavioral, and geographic variables. Syphilis rates were compared with rates of other common STIs (chlamydia and gonorrhea) to contextualize observed trends.

Chi-square tests were performed to assess associations between syphilis occurrence and categorical variables, including race/ethnicity, sex, substance use, and sexual behavior. Odds ratios (ORs) with corresponding 95% confidence intervals (CIs) were calculated for variables with available denominators. Statistical significance was set at a p-value < 0.05. Due to limitations of the CDC dataset, confounders such as socioeconomic status, education, or healthcare access could not be directly assessed.

### Data visualization

All visualizations were generated using the *ggplot2* package in R software. Bar charts with standard deviation error bars were created using geom_bar(stat="identity”) for categorical variables, and geom_errorbar() to depict variability. Geographic distribution maps were constructed using CDC-provided county-level data to illustrate state-specific syphilis prevalence, with darker shading representing higher case rates.

## Results

### Trends in common sexually transmitted infections in the United States, 2018–2022

**Fig. 1:**
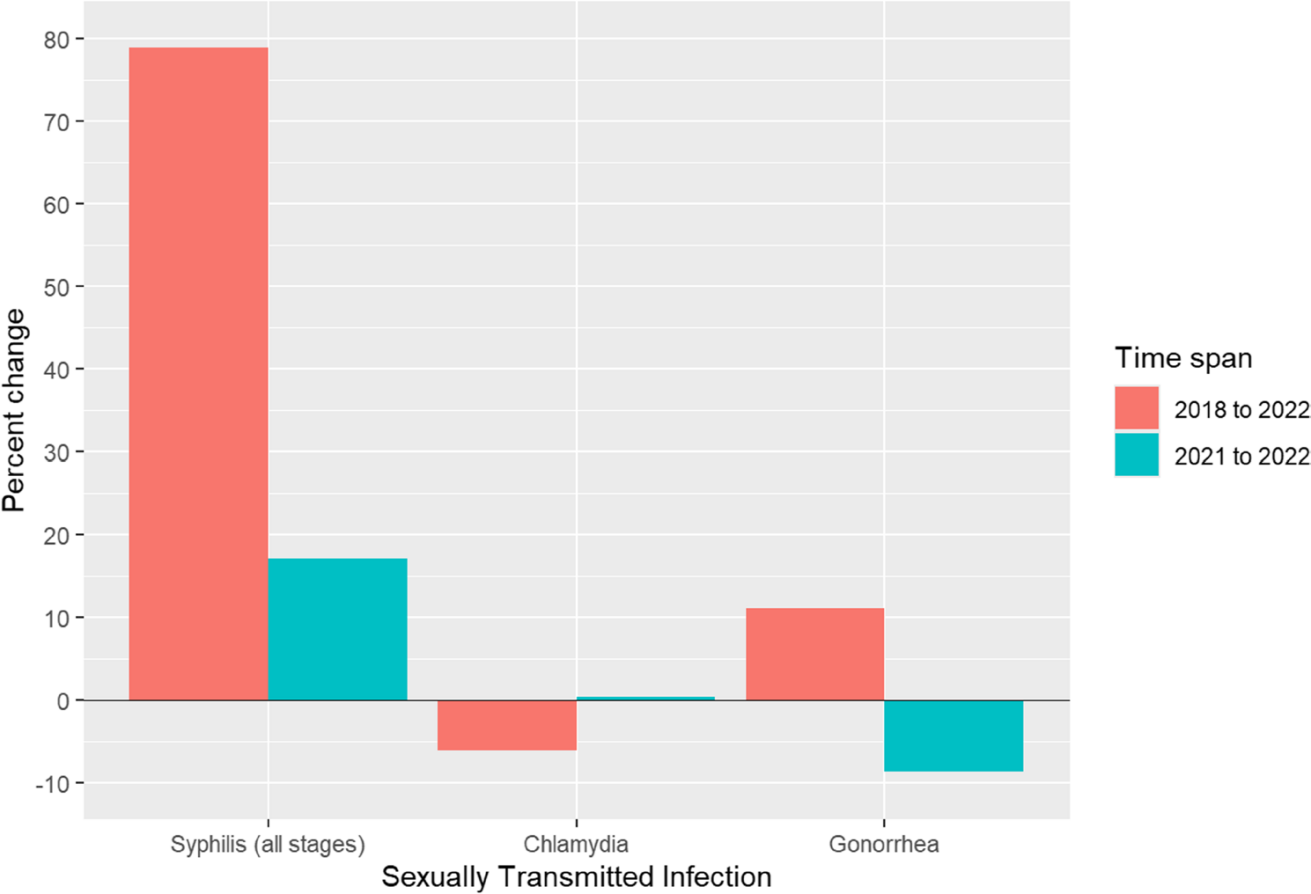
Percent change in reported cases of common Sexually Transmitted Infections (STIs) in the United States, 2018–2022 and 2021–2022

Recent epidemiological trends highlight a shifting and increasingly concerning burden of STIs across the United States. Chlamydia and gonorrhea continue to be among the most commonly reported communicable diseases nationwide. While chlamydia demonstrated an overall decline of 6.20% over the past five years, it remained relatively stable with a marginal increase of 0.30% in the most recent year (Fig. 1).

Gonorrhea, in contrast, exhibited a five-year increase of 11.10%, despite an 8.70% decrease from 2021 to 2022 (Fig. 1) Syphilis displayed the most dramatic change, with a 78.90% increase over five years and a 17.00% rise in just the last year alone (Fig. 1 and Supplementary Figure S1).

One-year (2021–2022) and five-year (2018–2022) percent changes in reported cases of chlamydia, gonorrhea, and syphilis based on CDC surveillance data. Percent change was calculated relative to case counts in 2018 and 2021, respectively. Syphilis demonstrated the most substantial increase over both time periods. Statistical significance of changes in syphilis rates was confirmed using a z-test for difference in proportions (*p* < 0.001 for both comparisons).

### Geographic distribution of syphilis cases across the United States

**Fig. 2:**
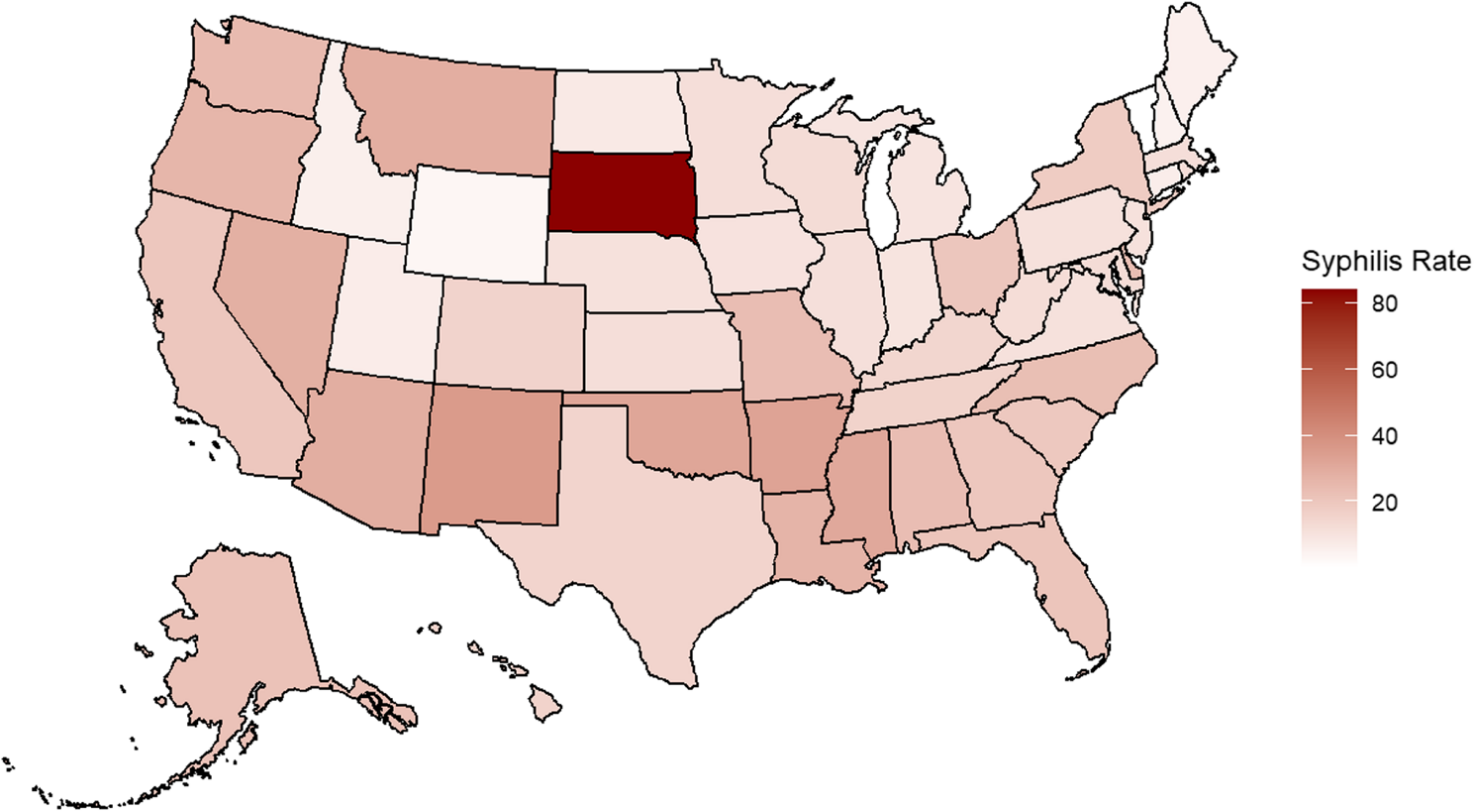
Geographic distribution of syphilis rates by State, United States, 2022

Geographic analysis revealed that the highest rates of syphilis including both primary and secondary syphilis as well as congenital syphilis, were concentrated in the Midwest and Southern regions of the United States (Fig. 2). Among these, South Dakota was disproportionately affected, reporting the highest rate of primary and secondary syphilis during the study period.

These findings underscore the need for targeted interventions that account for regional variations in resource availability, healthcare delivery, and social determinants of health.

Choropleth map illustrating primary and secondary syphilis rates by state in 2022 based on CDC surveillance data. Darker shading indicates higher rates of syphilis per 100,000 population.

### Sex- and age-related patterns of syphilis incidence

Between 2018 and 2022, syphilis cases were consistently higher among males compared to females, reflecting a greater reported burden of infection among males during this period (Fig. 5A)*.* While both sexes experienced an upward trend in case counts over the five-year span, males consistently accounted for a disproportionately greater number of cases. Notably, the number of syphilis cases among females nearly doubled, reflecting a concerning rise in incidence within this group as well.

**Fig. 3:**
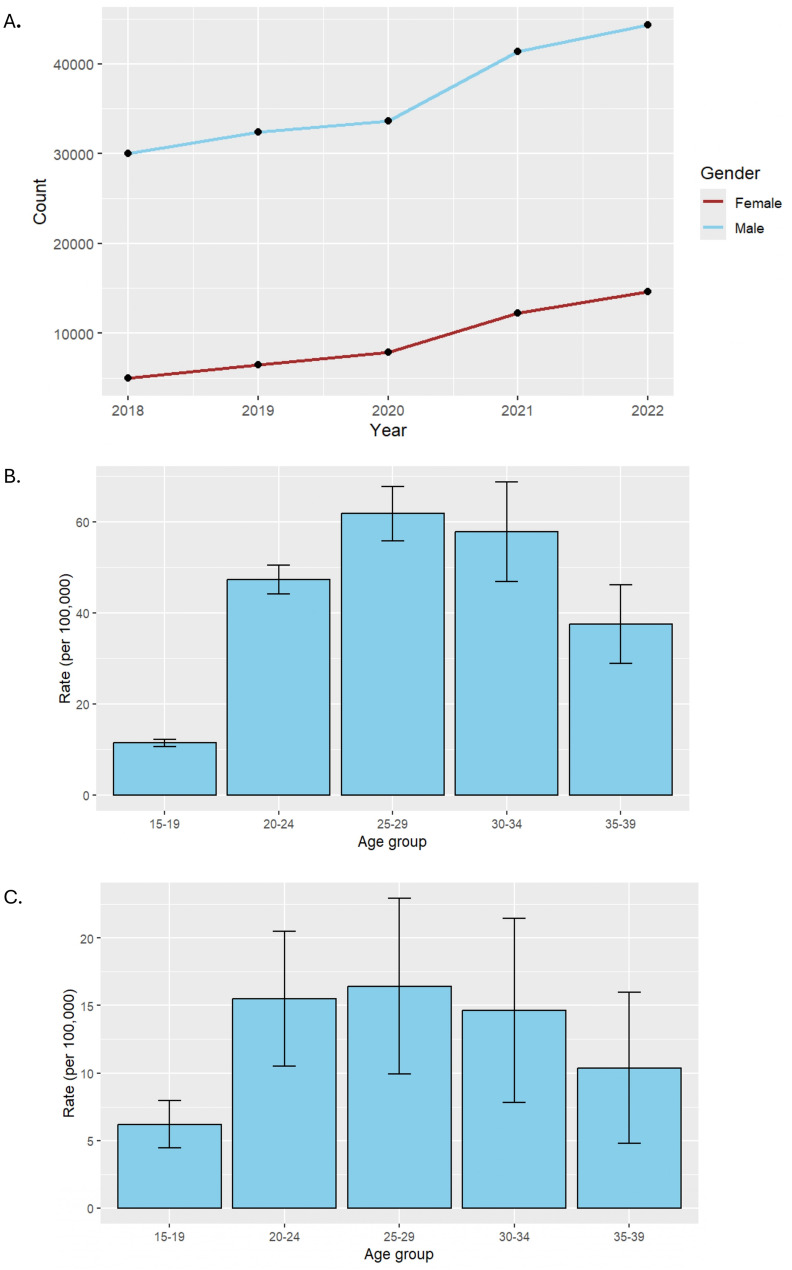
**A** Annual reported syphilis cases by sex, United States, 2018–2022. **B** Average Syphilis Rates Among Males by Age Group, United States, 2018–2022. **C** Average Syphilis Rates Among Females by Age Group, United States, 2018–2022. Trends in reported syphilis case counts from 2018 to 2022, stratified by sex. While males consistently accounted for a higher number of cases each year, the rate of increase among females was more pronounced over the five-year period, with reported cases nearly doubling from 2018 to 2022. *“Count”* on the Y-axis = *Number of reported P&S syphilis cases*. Average syphilis rates per 100,000 population among males, stratified by age group, from 2018 to 2022. Error bars represent the standard deviation across annual rates during the five-year period. Average syphilis rates per 100,000 population among females, stratified by age group, from 2018 to 2022. Error bars represent the standard deviation across annual rates during the five-year period

To further evaluate these trends, syphilis rates were averaged across the five-year period and stratified by age group for each sex. Among both males and females, the 25–29-year-old age group exhibited the highest rates of syphilis during the study period (Fig. 3B and C) In females, greater variability in case rates was observed beginning at age 20, as indicated by wider standard deviations, suggesting potential fluctuations in exposure or testing practices over time.

### Racial and ethnic disparities in syphilis rates

**Fig. 4:**
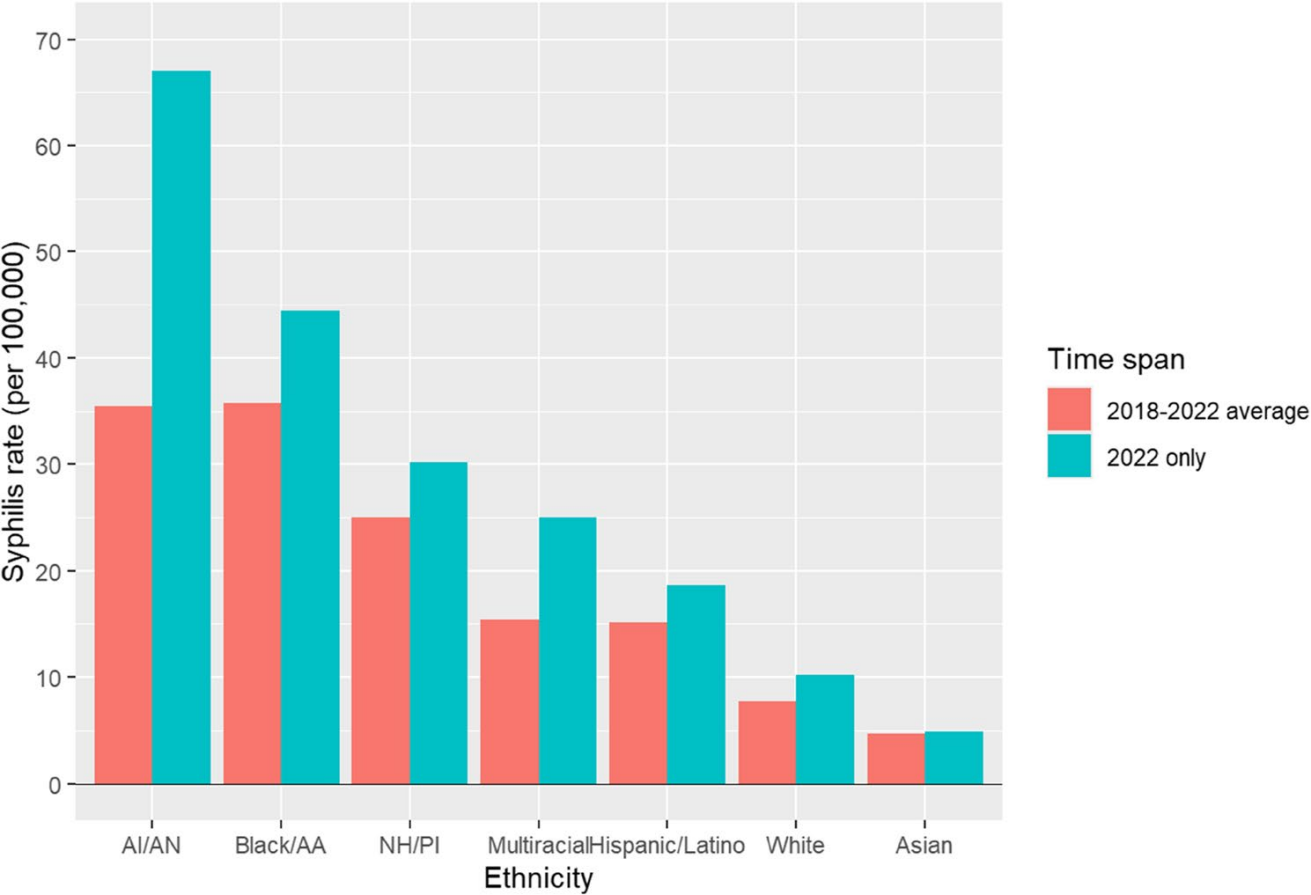
Syphilis rates by race and ethnicity, United States, 2018–2022. Rates of primary and secondary syphilis per 100,000 population among racial and ethnic groups. Bars represent the 2018–2022 average and 2022 rates separately. AI/AN = American Indian/Alaska Native; Black/AA = Black/African American; NH/PI = Native Hawaiian/Pacific Islander

While all racial and ethnic groups experienced increases in syphilis cases between 2018 and 2022, certain populations bore a disproportionately higher burden of infection. Individuals identifying as Black/African American and American Indian/Alaska Native (AI/AN) exhibited comparable five-year average rates of syphilis. However, in 2022, AI/AN individuals had the highest reported infection rate, which significantly influenced the overall average for this group (Fig. 4)*.*

Odds ratio analysis in 2022 demonstrated that AI/AN individuals had the highest likelihood of syphilis infection compared to White Americans (OR: 17.87; 95% CI: 14.01–22.80; *p* < 0.001), followed by Black/African Americans (OR: 7.03; 95% CI: 5.53–8.94; *p* < 0.001) (Table 1). All racial and ethnic groups showed significantly elevated odds of infection relative to White Americans, except those identifying as Asian, who had a significantly lower odds of infection (OR: 0.45; 95% CI: 0.32–0.65; *p* < 0.001).

**Table 1:** Odds ratios (OR) for syphilis infection by race and ethnicity, United States, 2022

Race/Ethnicity	Odds Ratio (OR)	95% Confidence Interval	*p*-value
American Indian/Alaska Native (AI/AN)	17.87	14.01–22.80	< 0.001
Black/African American (Black/AA)	7.03	5.53–8.94	< 0.001
Native Hawaiian/Pacific Islander (NH/PI)	3.81	2.98–4.87	< 0.001
Multiracial	2.93	2.29–3.77	< 0.001
Hispanic/Latino	2.01	1.55–2.61	< 0.001
White (Reference Group)	1.00	—	—
Asian	0.45	0.32–0.65	< 0.001

This table presents the odds ratio of syphilis for racial and ethnic demographics in 2022. White Americans were used as the reference group. All racial groups showed a higher likelihood of syphilis infection when compared to White Americans apart from Asian race

### Syphilis prevalence by sexual behavior characteristics

**Fig. 5:**
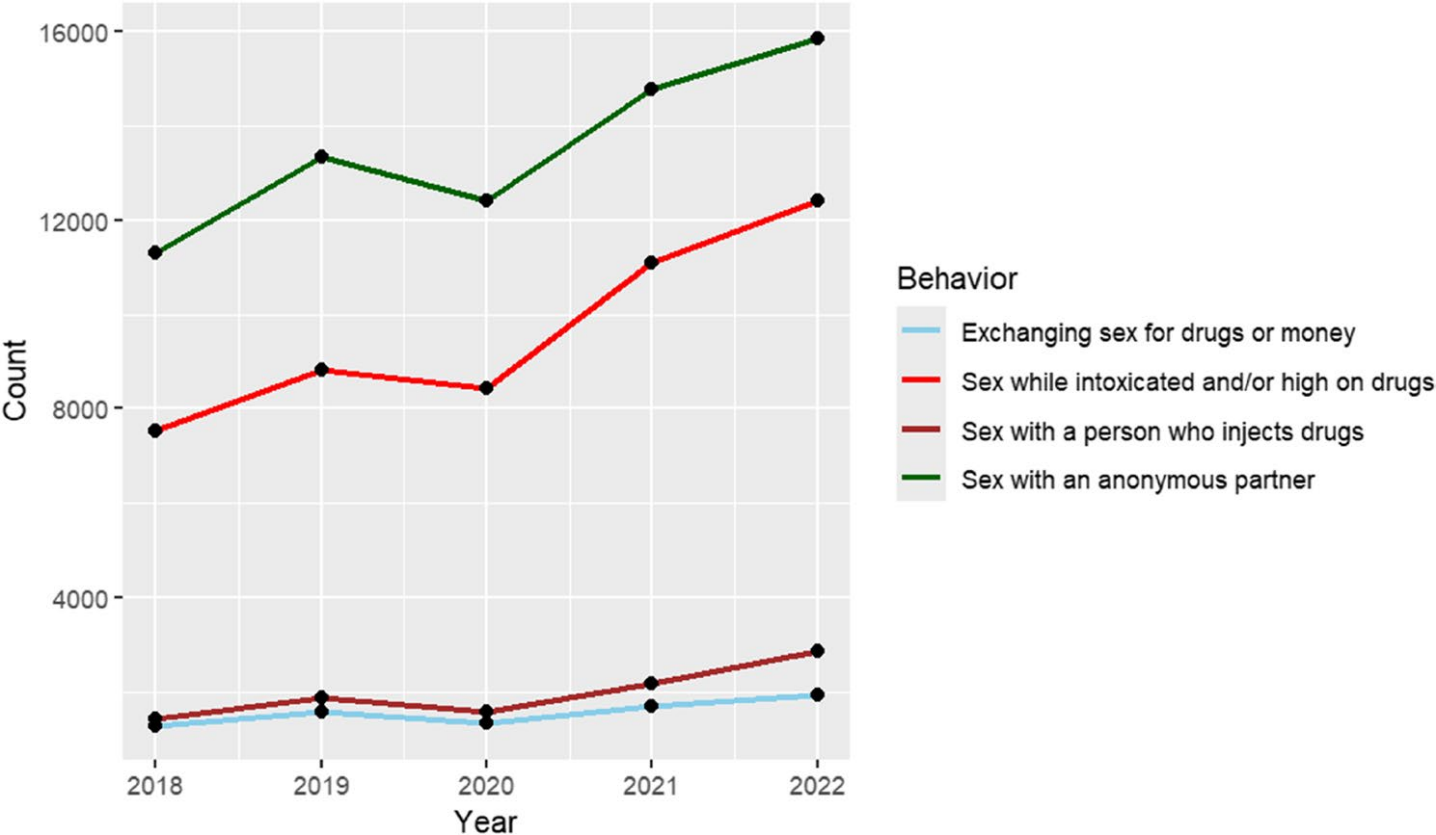
Syphilis cases by reported sexual behaviors, United States, 2018–2022. Total number of reported syphilis cases from 2018 to 2022, stratified by sexual behavior. Sexual behavior categories, as defined by the CDC, include exchanging sex for drugs or money, sex while intoxicated or high on drugs, sex with a person who injects drugs, and sex with an anonymous partner. *“Count”* on the Y-axis = *Number of reported P&S syphilis cases*

Between 2018 and 2022, individuals reporting sexual activity with anonymous partners consistently exhibited the highest rates of syphilis, with this behavior associated with the greatest risk of infection in 2022 (OR: 13.1; 95% CI: 9.70–17.72; *p* < 0.001) (Fig. 5; Table 2). Engaging in sexual activity while intoxicated was also strongly associated with increased risk (OR: 9.49; 95% CI: 6.99–12.83; *p* < 0.001). In comparison, having sex with a person who injects drugs was associated with a more modest increase in risk (OR: 1.68; 95% CI: 1.17–2.37; *p* < 0.001).

When evaluated against the reference category of transactional sex (defined as exchanging sex for drugs or money), all other behaviors, including anonymous sex, sex while intoxicated or high on drugs, and sex with a person who injects drugs, were associated with significantly higher odds of syphilis infection.

Moreover, all four behavior categories demonstrated a general upward trend in reported syphilis cases over the five-year period, with certain behaviors, particularly anonymous sexual activity and intoxicated sex, showing more pronounced increases (Fig. 5)*.*

**Table 2:** Odds ratios (OR) for syphilis infection by reported sexual behaviors, united states, 2022

Sexual Behavior	Odds Ratio (OR)	95% Confidence Interval	*p*-value
Exchanging sex for drugs or money (Reference Group)	1.00	—	—
Sex with an anonymous partner	13.10	9.70–17.72	< 0.001
Sex while intoxicated or high on drugs	9.49	6.99–12.83	< 0.001
Sex with a person who injects drugs	1.68	1.17–2.37	< 0.001

Odds ratios (OR) for syphilis infection in 2022 by reported sexual behavior, using exchanging sex for drugs or money as the reference group. All other behaviors were associated with significantly higher odds of syphilis infection. Engaging in sex with an anonymous partner showed the highest risk, with over 13 times greater odds of infection compared to the reference group

### Association between substance use and syphilis infection

**Fig. 6:**
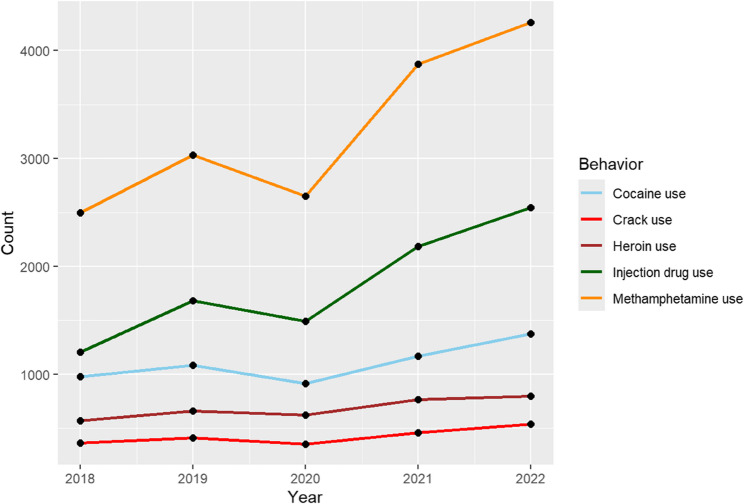
Syphilis rates by reported substance use, United States, 2018–2022. Average syphilis rates per 100,000 population from 2018 to 2022, stratified by reported substance use behavior. Substance use categories, as defined by the CDC, include cocaine, crack, heroin, injection drug use, and methamphetamine use. *“Count”* on the Y-axis = *Number of reported P&S syphilis cases*

All substance use behaviors depict an increasing trend over the five-year span from 2018 to 2022, further suggesting an increasing prevalence of syphilis (Fig. 6)*.* Individuals who use crack have 38% lower odds of syphilis infection compared to cocaine users (OR: 0.38, 95% CI: 0.35–0.42, *P* < 0.001) (Table 3). Similarly, heroin use is associated with 43% lower odds of syphilis infection (OR: 0.57, 95% CI: 0.52–0.62, *P* < 0.001). In contrast, injection drug use is linked to an 89% increase in the odds of syphilis infection (OR: 1.89, 95% CI: 1.77–2.02, *P* < 0.001). Methamphetamine use shows the strongest association, with users experiencing over three times higher odds of syphilis infection compared to cocaine users (OR: 3.34, 95% CI: 3.13–3.55, *P* < 0.001). These findings underscore the varying risks of syphilis infection among different substance users, with injection drug use and methamphetamine use being particularly associated with elevated risk.

**Table 3:** Syphilis OR for substance use in 2022

Substance Use Behavior	Odds Ratio (OR)	95% Confidence Interval	*p*-value
Cocaine use (Reference Group)	1.00	—	—
Crack use	0.38	0.35–0.42	< 0.001
Heroin use	0.57	0.52–0.62	< 0.001
Injection drug use	1.89	1.77–2.02	< 0.001
Methamphetamine use	3.34	3.13–3.55	< 0.001

Odds ratios (OR) for syphilis infection in 2022 by reported substance use behavior, using cocaine use as the reference group (OR = 1.00). Methamphetamine use was associated with the highest odds of syphilis infection, followed by injection drug use. In contrast, crack and heroin use were associated with significantly lower odds of infection compared to cocaine use

### Syphilis incidence by sex of sexual partner

**Fig. 7:**
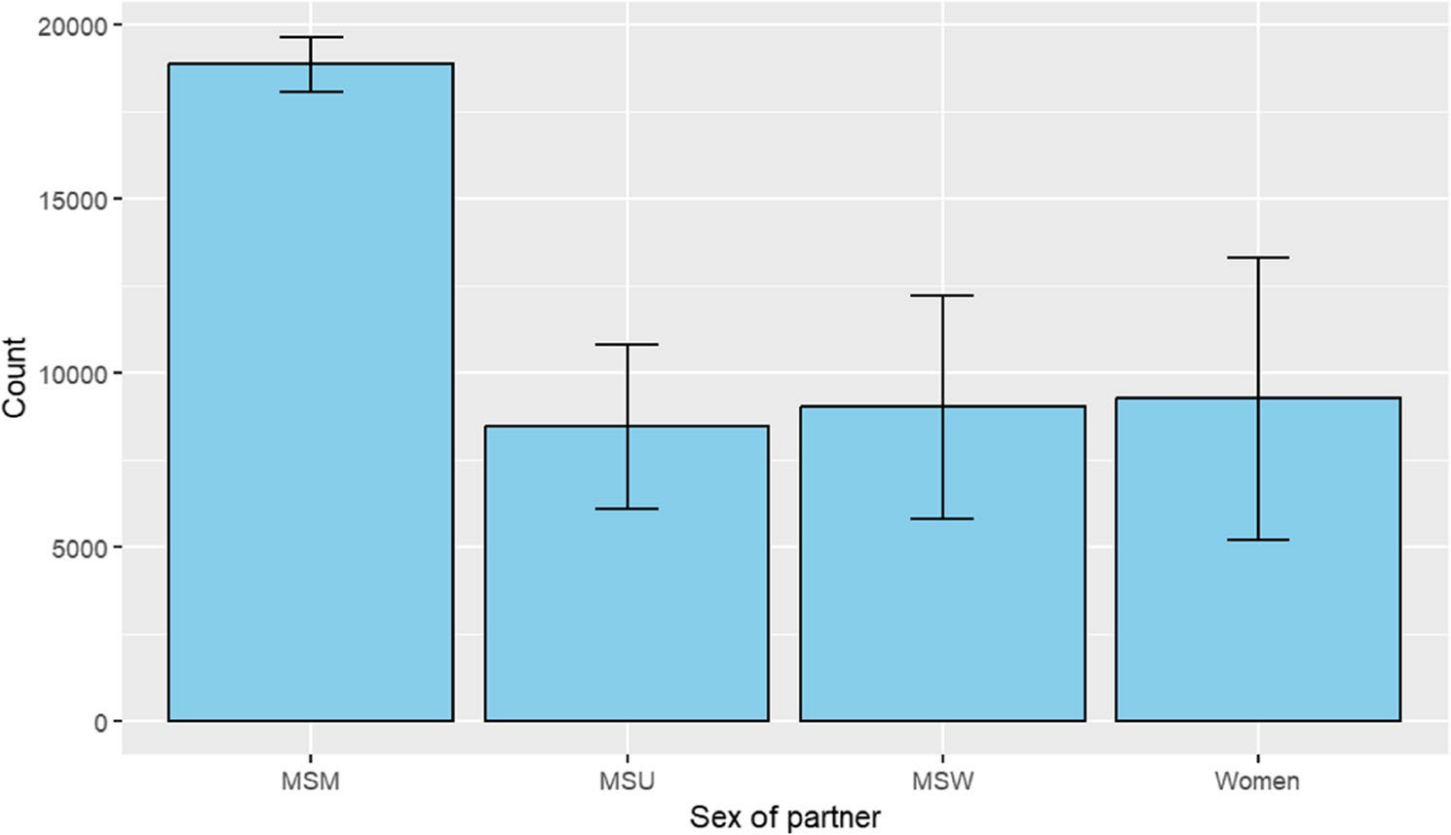
Total reported syphilis cases by sex of sexual partner, United States, 2018–2022. Total number of reported syphilis cases from 2018 to 2022, stratified by sex of sexual partner. Categories, as defined by the CDC, include men who have sex with men (MSM), men who have sex with women only (MSW), men with unknown sex of partners (MSU), and women. Unknown or missing data were excluded. Error bars represent the standard deviation across annual case counts during the five-year period. *“Count”* on the Y-axis = *Number of reported syphilis cases*

When stratified by reported sex of sexual partners, men who have sex with men (MSM) consistently accounted for the highest number of syphilis cases from 2018 to 2022 (Fig. 7)*.* While syphilis cases among MSM remained relatively stable over this period, greater year-to-year fluctuations were observed among men who have sex with women only (MSW), men with unknown sex of partners (MSU), and women. Notably, syphilis cases among women showed the greatest variability across the five-year period. The consistently high burden of syphilis among MSM underscores a persistent disparity in this population, which remains a critical focus for targeted public health interventions.

## Discussion

The resurgence of syphilis in the United States represents a growing public health crisis, driven by a complex interplay of social, structural, and behavioral determinants. Despite existing prevention efforts, including condom distribution and sex education programs, syphilis rates have risen sharply over the past five years, underscoring the limitations of current strategies in addressing this evolving epidemic [6]. These trends reflect a growing public health challenge, particularly in light of the resurgence of syphilis, and emphasize the need for enhanced surveillance, outreach, and intervention strategies.

Geographic and racial disparities were prominent, with the highest rates of syphilis reported in the Midwest and Southern regions, particularly in rural states like South Dakota, New Mexico, and Oklahoma. The identification of geographic hotspots for syphilis suggests potential disparities in healthcare access, public health infrastructure, and preventive services, particularly in rural and underserved areas. These findings underscore the need for targeted interventions that account for regional variations in resource availability, healthcare delivery, and social determinants of health. Our analysis shows that American Indian/Alaska Native (AI/AN) individuals were the most affected racial and ethnic group. In South Dakota, where AI/AN individuals make up a large proportion of the population, data indicate that this group accounted for a majority of syphilis cases during the resurgence [7, 8]. These patterns highlight the persistent healthcare disparities experienced by certain racial and ethnic groups, which may contribute to elevated rates of STIs. These areas face longstanding barriers to healthcare access, such as provider shortages, transportation limitations, and reduced availability of STI screening and treatment services [9, 10]. American Indian/Alaska Native (AI/AN) and Black/African American populations were disproportionately affected, reflecting historical and systemic inequities shaped by poverty, limited healthcare access, and systemic racism within healthcare delivery [11–14]. Strengthening partnerships with Indigenous-led healthcare organizations, expanding mobile clinics, and leveraging telemedicine services may help mitigate these barriers and improve access to STI prevention and care in high-burden communities. Previous community-engaged interventions, particularly in HIV prevention, have demonstrated the effectiveness of culturally tailored public health campaigns in improving testing uptake and reducing transmission in underserved populations [15, 16]. Such community-centered approaches should be applied to syphilis prevention efforts to ensure cultural relevance and trust-building within affected populations.

The widening disparities by sex and age highlight the need to expand prevention efforts beyond traditionally targeted populations. While males continue to bear a higher burden of syphilis, the near doubling of cases among females over the study period is concerning and may reflect under-testing, healthcare access barriers, and evolving behavioral risk patterns [17]. The marked increase in syphilis rates among individuals of reproductive age, particularly women of childbearing potential, likely contributed to the concurrent rise in congenital syphilis cases during this period. Integrating syphilis screening into routine reproductive health services, including prenatal care, and expanding outreach to women in high-burden areas should be prioritized.

Behavioral and substance use factors remain central drivers of syphilis transmission. High-risk sexual behaviors, including anonymous sex and sex while intoxicated or high on drugs, were significantly associated with increased odds of infection. One relevant consideration is the concept of “undetectable equals untransmissible” (U = U), which has gained prominence in the past decade as antiretroviral therapy has significantly improved viral suppression in people living with HIV [18]. As awareness and adoption of U = U have increased, some studies suggest a corresponding decline in condom use among individuals with HIV [18]. This behavioral shift may partially explain rising syphilis rates, particularly among men who have sex with men (MSM), individuals engaging in sex with anonymous partners, those using certain illicit drugs, and specific racial and ethnic groups. Additionally, methamphetamine use demonstrated the strongest association with syphilis, aligning with prior research linking stimulant use to increased sexual risk behaviors, particularly among men who have sex with men (MSM) [19]. Integrating STI prevention into harm reduction programs, including syringe service programs and substance use treatment services, is critical. Such approaches have been effective in reducing HIV and HCV transmission and should be adapted to address syphilis as well [15]. These findings indicate that while syphilis prevalence is increasing overall, the rate and intensity of this rise vary across demographic groups, reflecting underlying structural inequities and persistent barriers to care.

Beginning in 2020, syphilis rates stratified by sex, sexual behaviors, and substance use increased markedly compared to the period between 2018 and 2020. This surge aligns with the onset of the COVID-19 pandemic, which may have contributed to increased STI transmission due to social isolation, higher substance use, elevated engagement in high-risk sexual behaviors, and reduced access to healthcare services [20, 21]. Numerous studies have suggested that disruptions in diagnosis and treatment, resulting from the reallocation of healthcare resources during the pandemic, may have played a role in this increase [20, 21]. While more research is needed to establish a causal relationship, the temporal correlation between the COVID-19 pandemic and the rise in syphilis is notable.This study is not without limitations. Due to the constraints of the CDC dataset, key confounders such as socioeconomic status, education level, and healthcare access could not be directly assessed. The surveillance data used were derived from clinic-based reporting, which is subject to variability. Previous studies have identified underreporting of STIs and race/ethnicity data in private clinics compared to public health settings, potentially impacting the accuracy and completeness of surveillance data [22].Testing bias may also influence the interpretation of syphilis trends. For example, a retrospective analysis of patients seeking STI care found that men were more likely than women to receive comprehensive testing and to engage in additional preventive services such as HIV screening and risk-reduction counseling [22]. These patterns may help explain why men appear disproportionately affected, even as congenital syphilis rates continue to rise. In addition, research has shown racial disparities in STI testing practices, with Black individuals more likely to be tested for STIs compared to White individuals [16]. These biases should be taken into account when interpreting the observed disparities.

The recent shortage of benzathine penicillin G (BPG), the first-line treatment for syphilis, poses an additional barrier to disease control efforts [23]. Ensuring a stable and equitable supply of essential STI medications must be a public health priority, as treatment delays may lead to ongoing transmission and adverse health outcomes [24]. National policies aimed at strengthening the STI medication supply chain and creating reserve stockpiles could mitigate the impact of future shortages. Finally, improvements in STI surveillance infrastructure are urgently needed. Standardizing and improving the collection of race and ethnicity data, particularly in private healthcare settings, will enable more accurate monitoring of disparities and better allocation of resources [22, 25]. Targeted public health campaigns, such as the CDC’s successful “Get Yourself Tested” (GYT) initiative, have demonstrated the potential for increasing STI testing rates among youth and marginalized populations [26]. Leveraging similar culturally tailored, community-informed campaigns could enhance syphilis prevention efforts nationally.

## Conclusion

This study underscores that the resurgence of syphilis in the United States is neither random nor inevitable; it is a reflection of persistent health inequities shaped by geography, race, socioeconomic disadvantage, and behavioral health risks. The disproportionate burden of syphilis among American Indian/Alaska Native and Black/African American populations, individuals in rural areas, and those with substance use vulnerabilities signals critical gaps in current prevention and healthcare strategies.

Moving forward, a comprehensive public health response must go beyond clinical care alone. Integrating STI prevention with behavioral health services, expanding community-based screening and treatment, and investing in culturally tailored outreach are essential to reducing the burden of disease and narrowing health disparities. Strengthening these efforts will not only mitigate the growing impact of syphilis but also foster a more resilient, equitable, and responsive public health system.

## Supplementary Information

Supplementary Material 1.

Supplementary Material 2.

## Data Availability

The data supporting these findings are available for public access at the Centers for Disease Control and Prevention repository, https://www.cdc.gov/sti-statistics/.

## References

[1] Tudor ME. Syphilis. StatPearls. 2024. https://www.ncbi.nlm.nih.gov/books/NBK534780/.

[2] Centers for Disease Control and Prevention. Sexually transmitted infections surveillance. 2022. https://www.cdc.gov/sti-statistics. Accessed 30 Jan 2025.

[3] Centers for Disease Control and Prevention. Syphilis during pregnancy - STI treatment guidelines. 2021. https://www.cdc.gov/std/treatment-guidelines/syphilis-pregnancy.htm. Accessed 10 Apr 2025.

[4] Korenromp EL, Rowley J, Alonso M, et al. Global burden of maternal and congenital syphilis and associated adverse birth outcomes – Estimation using data from 2016 and 2012. PLoS ONE. 2019;14(2):e0211720. https://journals.plos.org/plosone/article?id=10.1371/journal.pone.0211720. 10.1371/journal.pone.0211720 PMC6392238 30811406

[5] Lafond RE, Lukehart SA. Biological basis for syphilis. Clin Microbiol Rev. 2006;19(1):29–49. 10.1128/CMR.19.1.29-49.2006. 16418521 10.1128/CMR.19.1.29-49.2006 PMC1360276

[6] Chen T, Wan B, Wang M, Lin S, Wu Y, Huang J. Evaluating the global, regional, and national impact of syphilis: results from the global burden of disease study 2019. Sci Rep. 2023;13: 11386. 10.1038/s41598-023-38294-4. 37452074 10.1038/s41598-023-38294-4 PMC10349077

[7] Great Planes Tribal Leaders’ Health Board. Sexually Transmitted infections. https://www.greatplainstribalhealth.org/sexually-transmitted-infections.html. Accessed 20 May 2025.

[8] Indian Health Services. Syphilis Statistics. https://www.ihs.gov/sti/syphilis/syphilisstatistics/. Accessed 20 May 2025.

[9] Schmidt R, Carson PJ, Jansen RJ. Resurgence of syphilis in the United States: an assessment of contributing factors. Infect Dis. 2019. 10.1177/1178633719883282. 10.1177/1178633719883282 PMC6798162 31666795

[10] Skewes MC, Blume AW. Understanding the link between racial trauma and substance use among American Indians. Am Psychol. 2019;74(1):88–100. 10.1037/amp0000331. 30652902 10.1037/amp0000331 PMC6338088

[11] Boutrin MC, Williams DR. What racism has to do with it: understanding and reducing sexually transmitted diseases in youth of color. Healthcare. 2021;9(6): 673. 10.3390/healthcare9060673. 34199974 10.3390/healthcare9060673 PMC8227416

[12] Fowler E. Addressing health disparities in [internet]ndian country during COVID-19: April 2021 blogs [Internet]. Indian Health Service Blog. 2021. [Cited 2025]. https://www.ihs.gov/newsroom/ihs-blog/april2021/addressing-health-disparities-in-indian-country-during-covid-19/.

[13] Leston J, Wenger H, Reilley B, Craig Rushing S, Rink E, Warren H, Howe J, Bloomquist P, Tah T, Jeffries I, Iralu J, Thorpe P, Apostolou A, Taylor MM. Creating a path forward: understanding the context of sexual health and sexually transmitted infections in American Indian/Alaska Native populations – a review. Sex Health. 2022;19(4):286–98. 10.1071/SH22040. 35760766 10.1071/SH22040 PMC11081199

[14] Sarche M, Spicer P. Poverty and health disparities for American Indian and Alaska native children. Ann NY Acad Sci, 2008;1136(1):126–136. 10.1196/annals.1425.017. 10.1196/annals.1425.017 PMC2567901 18579879

[15] Syringe Service Programs for Persons Who Inject Drugs in Urban. Suburban, and Rural Areas — United States, 2013. The Centers for Disease Control and Prevention; 2015. [cited 13 APR 2025]. Available from https://www.cdc.gov/mmwr/preview/mmwrhtml/mm6448a3.htm. 10.15585/mmwr.mm6448a3 26655918

[16] Vitsupakorn S, Pierce N, Ritchwood TD. Cultural interventions addressing disparities in the HIV prevention and treatment cascade among black/african americans: a scoping review. BMC Public Health. 2023;23(1):1748. 10.1186/s12889-023-16658-9. https://bmcpublichealth.biomedcentral.com/articles/. 37679765 10.1186/s12889-023-16658-9 PMC10485990

[17] Yumori C, Zucker J, Theodore D, Chang M, Carnevale C, Slowikowski J, LaSota E, Olender S, Gordon P, Cohall A, Sobieszczyk ME. Women are less likely to be tested for HIV or offered preexposure prophylaxis at the time of sexually transmitted infection diagnosis. Sex Transm Dis. 2021;48(1):32–6. 10.1097/OLQ.0000000000001265. 33315784 10.1097/OLQ.0000000000001265 PMC8543120

[18] Eisinger WR, Dieffenbach WC, Fauci SA. HIV viral load and transmissibility of HIV infection undetectable equals untransmittable. JAMA. 2019;321(5):451–2. https://jamanetwork.com/journals/jama/article-abstract/2720997. 30629090 10.1001/jama.2018.21167

[19] Hirshfield S, Remien RH, Walavalkar I, Chiasson MA. Crystal methamphetamine use predicts incident STD infection among men who have sex with men recruited online: a nested case-control study. J Med Internet Res, 2024;6(4):e41. 10.2196/jmir.6.4.e41. 10.2196/jmir.6.4.e41 PMC1550619 15631965

[20] Liu M, Zhou J, Lan Y, Zhang H, Wu M, Leng L, Mi X, Li J. A neglected narrative in the COVID-19 pandemic: epidemiological and clinical impacts of the COVID-19 outbreak on syphilis. Clin Cosmet Invest Dermatology. 2023;16:2485–96. https://www.dovepress.com/a-neglected-narrative-in-the-covid-19-pandemic-epidemiological-and-cli-peer-reviewed-fulltext-article-CCID#cit0015. 10.2147/CCID.S417522 PMC10505047 37719933

[21] Wright S, Kreisel K, Hitt J, Pagaoa M, Weinstock H, Thorpe P. Impact of the COVID-19 pandemic on centers for disease control and Prevention–Funded sexually transmitted disease programs. Sex Transm Dis. 2022;49(4):e61–3. https://pmc.ncbi.nlm.nih.gov/articles/PMC9214625/. 34654769 10.1097/OLQ.0000000000001566 PMC9214625

[22] Ross MW, Courtney P, Dennison J, Risser JM. Incomplete reporting of race and ethnicity in gonorrhoea cases and potential bias in disease reporting by private and public sector providers. Int J STD AIDS. 2004;15(11):778. 10.1258/0956462042395168. 15537469 10.1258/0956462042395168

[23] Nelson R. Syphilis rates soar in the USA amid penicillin shortage. Lancet (London England). 2023;402(10401):515. 10.1016/S0140-6736(23)01665-3. 37573855 10.1016/S0140-6736(23)01665-3

[24] Snider W, Depew I, Cook S, Roth D, Benzathine Penicillin G, Shortage. Secondary Syphilis Cureus. 2024;16(8):e66787. 10.7759/cureus.66787. 39268312 10.7759/cureus.66787 PMC11392051

[25] Rolin A, Badolato G, Doyle M, Foltz D, Goyal M. Racial differences in sexually transmitted infection testing in adolescent males presenting to the emergency department. J Adolesc Health. 2025;76(3). 10.1016/j.jadohealth.2024.11.141.

[26] Centers for Disease Control and Prevention (CDC). Get Yourself Tested (GYT) Campaign Overview. 2017. https://www.cdc.gov/sti/?CDC_AAref_Val=https://www.cdc.gov/std/prevention/gyt.htm. Accessed 2025.

