# Supplementary material for: Epidemiological analysis of syphilis trends, disparities, and public health implications in the United States, 2018–2022
Source: BMC Infect Dis. 2025 Sep 12;25:1106. doi: 10.1186/s12879-025-11332-4 (PMC12427103; doi:10.1186/s12879-025-11332-4)

**Supplementary Figure 1. Trends in Primary and Secondary Syphilis Rates, United States, 2018–2022.**

Rates of primary and secondary syphilis per 100,000 population from 2018 to 2022, based on CDC surveillance data. The figure illustrates a substantial increase in syphilis rates over the five-year period.


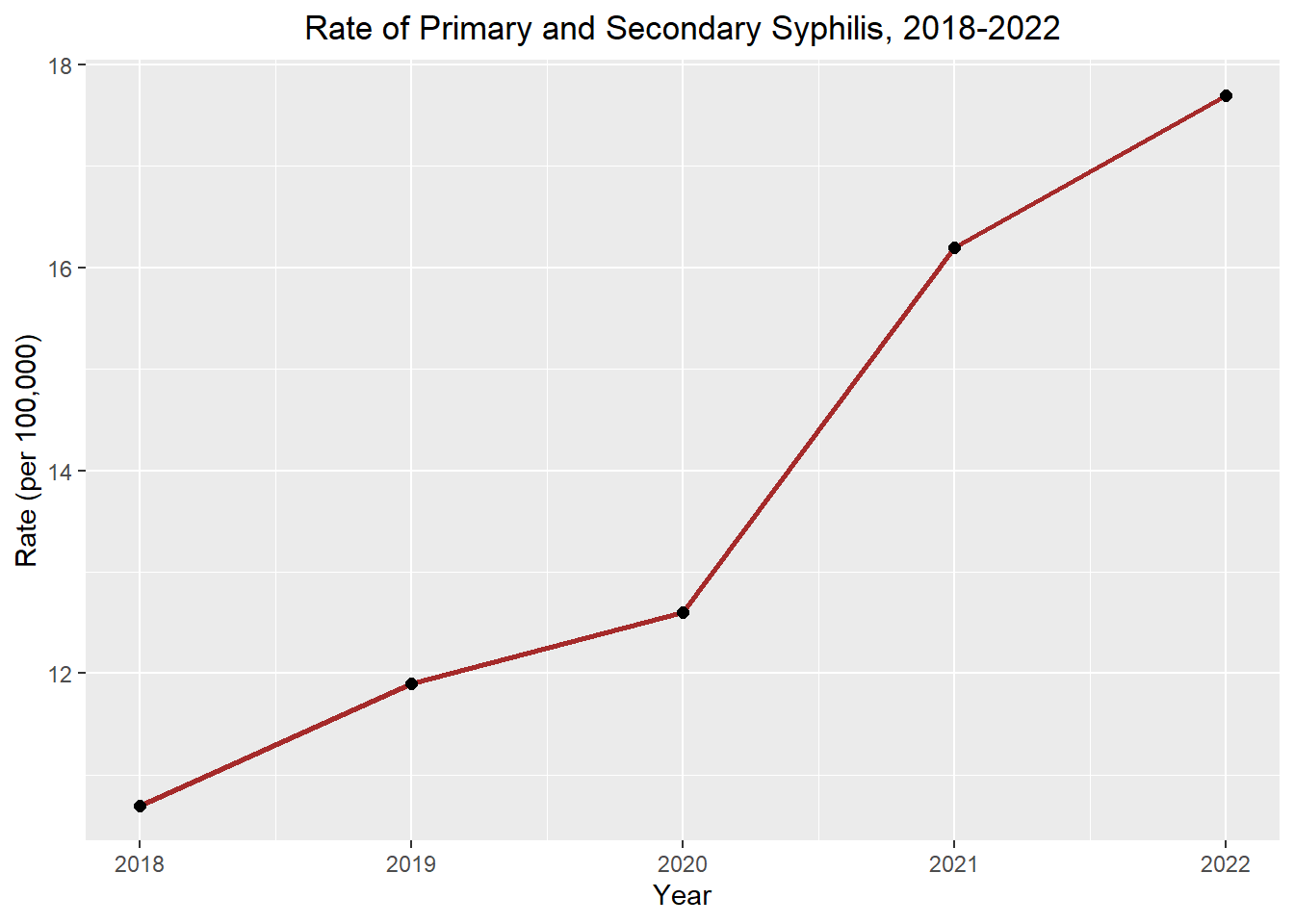

Supplement: Supplementary file 2 — Supplementary Material 2. [file 12879_2025_11332_MOESM2_ESM.docx]
